# Yield of tuberculosis among household contacts of tuberculosis patients in Accra, Ghana

**DOI:** 10.1186/s40249-018-0396-5

**Published:** 2018-02-27

**Authors:** Sally-Ann Ohene, Frank Bonsu, Nii Nortey Hanson-Nortey, Adelaide Sackey, Samuel Danso, Felix Afutu, Paul Klatser, Mirjam Bakker

**Affiliations:** 1 World Health Organization Country Office, 29 Volta Street Airport, Airport Residential Area, P.O. Box MB 142, Accra, Ghana; 2 National Tuberculosis Control Program, Accra, Ghana; 3 Department of Global Health, Academic Medical Centre, Amsterdam Institute of Global Health and Development, Amsterdam, The Netherlands; 4 KIT Health, Royal Tropical Institute (KIT), Amsterdam, The Netherlands

**Keywords:** Ghana, Tuberculosis, Tuberculosis contact investigation, Screening

## Abstract

**Background:**

The End TB Strategy calls for systematic screening of selected high-risk groups including contacts of tuberculosis (TB) cases to facilitate early TB case detection. Contact investigation is not usually routinely practiced in low TB burden countries, such as Ghana, with consequent paucity of data on the yield of TB case detection from such interventions. This study’s objective was to document the outcomes and feasibility of implementing contact investigation activities under programmatic conditions in Ghana.

**Methods:**

Retrospective analyses were conducted of abstracted data from the National TB Program, following a contact investigation intervention for TB cases diagnosed in 10 facilities in Accra from June 2010 to December 2014. Various proportions and yield from number of contacts needed to screen (NNS) and number needed to test (NNT) to detect a TB case were assessed.

**Results:**

Overall, out of the 8519 listed contacts of 3267 index cases, 8166 (96%) were screened and 614 (7.5%) were identified as presumptive TB. Out of these, 438 (71%) underwent sputum smear microscopy/evaluation and 53 TB cases were diagnosed. Of these, 56.6% were males, and 49% had sputum smear-positive TB, 38% had sputum smear-negative TB, and 7% had extra-pulmonary TB. The NNS and NNT to detect a TB case of all forms were 154 and 8, respectively. The proportion of TB cases with contacts listed and proportion of contacts screened annually were 88–96% and 83–100%, respectively. The proportion of presumptive TB cases tested and proportion of TB cases diagnosed among contacts tested that were 100% and 36%, respectively, in 2010 dropped to 40% and 14%, respectively, by 2014.

**Conclusions:**

The study demonstrates that contact identification and prioritization components of a contact investigation were feasible, but overall yield of TB cases may have been lower due to the declining rate of clinical evaluation of presumptive TB contacts over time. Addressing barriers to accessing appropriate diagnostic tests may enhance yield from contact investigation in Ghana.

**Electronic supplementary material:**

The online version of this article (10.1186/s40249-018-0396-5) contains supplementary material, which is available to authorized users.

## Multilingual abstracts

Please see Additional file 1 for translations of the abstract into six official working languages of the United Nations.

## Background

Tuberculosis (TB) remains a disease of public health concern globally. The World Health Organization (*WHO) Glo*bal TB Report 2016 shows that *in 2015, there were an estimated 10.4 million new TB cases worldwide* [1]*. A significant portion of this figure, more than 40% (about 4.3 million), is however not notified.* This pool of unidentified TB cases poses a risk for further transmission of TB. Consequently to ensure that this high burden of undiagnosed TB is reduced, it is imperative that strategies that enhance TB case detection with subsequent appropriate treatment are scaled up.

Contact investigation has been proposed as a worthwhile strategy to enhance early detection of TB cases and reduce transmission in high incidence localities [2, 3]. Contacts of TB cases are at a higher risk of acquiring TB than the general population because of their direct exposure to airborne droplets containing *Mycobacterium tuberculosis* [4]. Reviews of studies from low- and middle-income settings looking at contact investigation for TB have reported the prevalence of active TB in all contacts to be in the range of 3.1–4.5% and that for microbiologically-proven TB to be 1.2–2.3% [2, 4]. While in high-income countries, contact investigation is common practice, the same cannot be said for lower-income settings, where resource constraints and inadequate procedures pose as challenges [3]. Furthermore, given the paucity of data on the yield and contribution of TB case detection from contact investigation in these settings, the ability to assess the contribution of implementing this intervention in reducing transmission in these countries is also limited [3].

One of the challenges identified in the 2009–2013 National Tuberculosis Control Program (NTP) Strategic Plan was the low TB case detection rate in Ghana, estimated at 36% of pulmonary smear positive cases [5]. Therefore, improving case detection was targeted as an area for intervention using different innovative strategies. Investigation of contacts of TB cases was among the activities identified to be implemented in the Ghana TB strategic plan, as there was no structured and systematic way of conducting contact tracing in the country. In 2009, the WHO with funding from the Canadian International Development Agency (CIDA) supported a number of case detection initiatives implemented by the NTP in health facilities in the Accra metropolis in Ghana [6, 7]. Among them was a TB contact investigation component, which was initiated in 2010 and supported until 2013.

Given the dearth of data on the subject in a number of African countries and Ghana in particular, the objective of this paper is to document the outcomes of the contact investigation activities implemented in an urban area, highlighting the feasibility of implementing such an intervention under programmatic conditions.

## Methods

### Study design and setting

This study is a retrospective analysis of the performance of a contact investigation intervention implemented by the NTP in 10 facilities in Accra, the capital of Ghana, which has a population of 1.5 million.

Tuberculosis control in Accra, as in the rest of the country, is supported by the NTP, which has the mandate of leading the health sector response to TB control [5]. The NTP staff at the national level comprising the program manager and technical officers liaise with TB coordinators and multidisciplinary TB teams at subnational levels to facilitate TB control activities. At community and facility levels, there is a dedicated TB focal person who is supported by community health workers, volunteers, and treatment supporters to implement activities including registration, home verification, management, and follow-up of TB patients. The control of TB is integrated into all levels of care with 100% directly observed treatment, short-course (DOTS) coverage.

In the years preceding the initiative, the TB case notification rate ranged from 57 to 64 per 100 000, with treatment success reaching 85% [6]. Sputum smear microscopy was the mainstay of TB diagnosis, though steps were being taken to make culture services available. GeneXpert had also not been introduced for TB diagnosis at that time. Case detection was mainly passive with symptomatic patients self-reporting at health facilities.

Before the introduction of the WHO/CIDA supported case finding initiative, there was no structured or systematic way of conducting contact tracing. As a result, systems put in place by the NTP to facilitate this exercise included the development of operating guidelines for conducting contact investigation, a contact investigation register, a questionnaire for screening of contacts, and a monthly contact investigation reporting form that the staff submitted to the NTP. Staff members in the participating facilities were also trained in the contact investigation operating guidelines to undertake the exercise.

Following the identification of an index TB case, defined in the guidelines as a confirmed patient with sputum smear-positive (SS + ve) TB, sputum smear-negative (SS-ve) TB, or extra-pulmonary TB (EPTB), the person was requested to list all their contacts. The definition of a contact was derived from what was used in the Ghana Demographic and Health Survey: namely, persons living together in the same house sharing the same housekeeping arrangements and eating together [8]. With the consent of the index patient, the designated contact investigation focal person at the facility, usually a community health officer, visited the home at an agreed time with the list of contacts to do conduct screening for. The index patient also had the option of coming to the health facility with the contacts to be screened. Using a questionnaire that asked for symptoms of cough, fever, drenching night sweats, and weight loss, the contacts were screened and assigned a score depending on the responses given. Those who were suspected to have TB were requested to undergo sputum smear examination at the nearest facility. In these cases, two specimens of sputum were collected, one on the spot and the other at least one hour later or early in the morning within 24 h, for examination under a microscope for acid fast bacilli (AFB). A diagnosis of SS + ve TB was made if at least one sample was found to be positive for AFB, defined as at least one AFB identified in 100 fields. A clinician diagnosed SS-ve TB after clinical examination and X-ray findings suggestive of TB, following SS-ve microscopy results. Contacts who were subsequently diagnosed with TB were referred for treatment at the nearest TB DOTS facility. In households with contacts aged under five years, the children were referred to an experienced clinician or a pediatrician for a thorough examination. If active TB disease was confirmed, the child would be put on full-course anti-TB treatment. The guidelines indicated that those children who were not found to have active TB disease were to be put on isoniazid prophylaxis for six months at a dose of 10 mg/kg.

### Data collection and analyses

The data repository of the NTP was the key source of data for these analyses. The following data, which each facility conducting contact investigation submitted on a monthly basis to the NTP, were compiled into annual data: the number of index cases identified and those for whom contacts were listed, the number of contacts listed, the number of those listed who were screened, the number identified as presumed TB among those screened, and the number tested and diagnosed with TB. The data covered the period from June 2010 to December 2013, as well as 2014 during which funding for the project officially ceased, but facilities continued contact investigation of index cases.

Among those diagnosed with TB, the sex of the patient and the type of TB were also reported (SS + ve, SS-ve, or EPTB). Children who could not undergo smear microscopy but who were diagnosed clinically as having TB had their type of TB categorized as ‘other’. There was no age variable on the reporting form and therefore the ages of the contacts who were diagnosed as TB patients were not available for analyses.

The key outcome of the analyses was the prevalence of TB cases among contacts of index TB patients. The following indicators were assessed: proportion of index cases for whom contacts were listed, proportion of contacts listed screened for TB, proportion of contacts suspected to have TB (also referred to as presumed TB contacts), and proportion of presumed TB contacts who underwent sputum smear microscopy. The number of contacts needed to screen (NNS) to identify a TB case (contacts screened/new cases) overall and per year, and the number of contacts needed to test (NNT) to identify a TB cases (contacts tested/new cases) were also calculated. Using the chi-square test, the types of TB in terms of sex were analyzed. Statistical significance was defined as *P*-value < 0.05.

The Ministry of Health Research Division Ethical Review Board gave ethical clearance for the study, while the NTP granted permission for the use of data. Confidentiality was maintained in the conduct of the analyses.

## Results

**Fig. 1:**
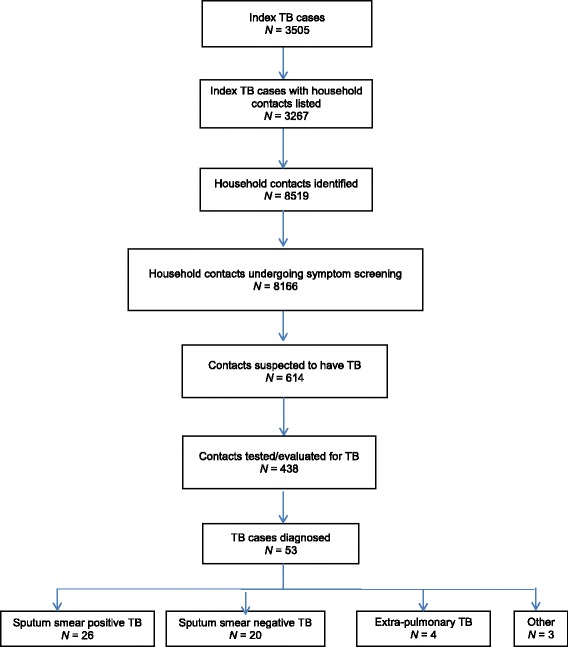
Flow diagram showing outcome of contact investigation 2010–2014, Accra

From June 2010 to December 2014, 3505 index TB patients were identified from the facilities participating in the contact investigation (Fig. 1). Contact listing was undertaken for 3267 (93%) patients.

On average, 2.5 contacts were listed per index patient. Approximately 96% of the contacts listed (8166) were screened using the questionnaire and 614 were suspected to have TB (7.5%). Of these, 71% underwent sputum smear microscopy were evaluated for TB, with 53 cases of TB being diagnosed, representing 12.2% of those evaluated. Overall, the prevalence of TB among the contacts screened was 0.65%. To identify one TB case, the NNS was 154 and the NNT was 8.

**Fig. 2:**
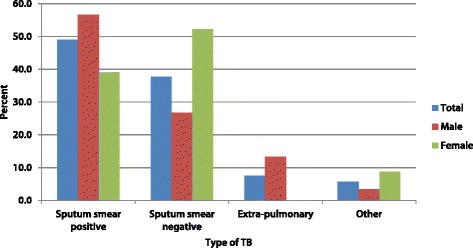
Type of TB diagnosed among contacts of index TB patients by gender

Among the contacts diagnosed with TB, 30 (56.6%) were males. Almost half (49%) were SS + ve TB cases, representing 5.9% of the contacts tested/evaluated for TB. Sputum smear was negative in 38% of the contacts diagnosed with TB while 7% had extra-pulmonary TB. More males than females had SS + ve TB, while reversely more females than males had SS-ve TB (Fig. 2). The differences across sex, however, were not statistically significant. All four EPTB cases diagnosed were males. Three of the cases (5.7%) were clinically diagnosed TB.

Compared to the other years, there was a relatively smaller number of index TB cases in 2010, as the intervention started in June 2010 (Table 1).

**Table 1:** Outcome of TB contact investigation in Accra, 2010–2014

Year	Number of index TB cases registered	Number of index cases with contacts listed	Number of contacts listed	Number of contacts screened	Number of contacts suspected to have TB	Number of contacts suspected with TB who were tested	TB cases identified	Prevalence of TB among contacts screened (%)	NNS	NNT
2010^a^	359	316	811	679	28	28	10	1.5	68	2.8
2011	873	826	2051	1953	136	136	15	0.8	130	9
2012	872	794	2285	2219	175	153	11	0.5	202	13.9
2013	797	749	1930	1945	139	64	9	0.5	216	7
2014^b^	604	582	1442	1370	136	57	8	0.6	171	7
Total	3505	3267	8519	8166	614	438	53	0.65	154	8

^a^The data from 2010 reflects only seven months of that year, from June to December. ^b^In 2014, contact investigation activities continued for the entire year up to December 31 even though funding for the project ceased

The number of contacts suspected to have TB was also comparatively smaller in 2010, however, the yield of TB cases among the contacts screened was the highest. The highest number of contacts listed, screened, and tested for TB was in 2012.

**Fig. 3:**
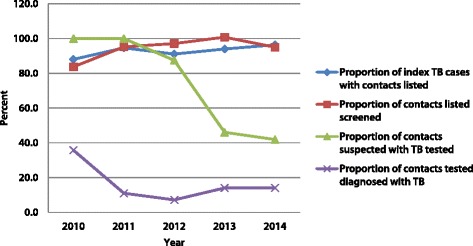
Performance of contact investigation conducted among index TB patients in Accra from 2010 to 2014

The proportion of TB cases with contacts listed each year was stable over the course of the five years, ranging from 88% to 96% (Fig. 3). Starting from 100%, the proportion of contacts suspected to have TB who were tested dropped by more than half to 40% in 2014. The year 2010 recorded the highest proportion of TB cases diagnosed among the contacts tested (36%).

## Discussion

This study reviewing a five-year contact investigation intervention conducted in Accra among index TB patients showed that contacts were listed for more than nine out of ten index TB cases. All but 4% of the contacts listed underwent symptomatic screening. Among those screened, seven out of ten of those suspected to have TB underwent sputum smear microscopy, ultimately yielding a TB prevalence of 0.65% among the contacts screened.

The average number of contacts identified per index TB case in our study was consistent with what others have found [4, 9, 10]. While other studies have reported higher figures for contacts identified per index TB case, it is worthwhile to note that various definitions of index cases and household contacts may account for the discrepancy of figures reported in different studies [3, 4, 11–13]. In our study, the definition of a household contact did not specify the period that the contact was in close proximity to the index case and it is not known whether this had an effect on the outcome. The consensus definition of household contacts is “a person who shared the same enclosed living space for one or more nights or for frequent or extended periods during the day with the index case during the 3 months before commencement of the current treatment episode” [3]. For standardization and comparability, it is recommended that TB control programs apply this definition to enhance monitoring and evaluation, and contribute to evidence on the impact of contact investigations [3].

Over the course of the contact investigation initiative, the proportion of listed contacts who had symptom screening, identified as presumptive TB and eventually tested for TB, fell within what was found in studies conducted in other countries [10, 14, 15]. Contact investigation of TB patients has been shown to identify significant numbers of TB cases in various contexts and improve TB case detection depending on the diagnostic tools used [9, 15, 16]. Comparative meta-analyses of TB case finding interventions in several low- and middle-resource high burden TB countries using sputum smear microscopy to diagnose TB showed that contact investigation interventions contributed from 0.1% to 14.2% of notified SS + ve TB cases, with a yield of 0.6% to 4.8% of all forms of TB diagnosed through contact investigation [15]. The yield from our study, which also used sputum smear microscopy as the main diagnostic procedure, falls within this range, even though Ghana is a low burden TB country. Blok et al. showed that the use of less restrictive criteria for identifying those eligible for sputum examination was associated with a higher yield of TB cases among contacts [15].

Nevertheless, the use of symptom screening and the low sensitivity of sputum smear microscopy in the diagnosis of TB limit the opportunity to detect TB early in high-risk groups, including contacts. This highlights the importance of promoting more sensitive screening tools, such as X-ray, to identify those who need to be further investigated for TB [17, 18]. The use of more sensitive diagnostic methods beyond conventional microscopy further improves the yield of TB cases among those suspected to have TB. It is therefore possible that the yield observed in our study may also be related to the use of conventional microscopy, which has relatively lower sensitivity than other TB diagnostic methods, yet was the mainstay of TB diagnosis at the time [19].

In consonance with other studies, the proportion of males and females among the contacts identified with TB were similar [16, 20]. While in our study the proportion of SS + ve and SS-ve TB cases were similar, Gashu and colleagues found that SS-ve TB was predominant among contacts diagnosed with TB in Ethiopia [11]. Blok et al. found that SS + ve index cases correlated with a higher percentage yield of SS + ve contacts compared to SS-ve index cases [15]. It is therefore plausible that there may have been a relatively higher proportion of SS + ve cases among the index patients in our study and this possibly explains the proportion of SS + ve cases among the contacts. Shapiro et al. also found a higher proportion of SS-ve cases in South Africa, but all these patients were found to be culture positive, highlighting the importance of using more sensitive tests such as culture or GeneXpert to improve detection of SS-ve TB, in cases where smear microscopy results are negative [16, 21].

The additional benefit of these tests is the ability to identify drug-resistant TB (DR-TB) [19]. While DR-TB cases had been identified at the time of the study in the country, the prevalence was not known. Given that TB in contacts of DR-TB cases is also likely to be drug resistant, the availability of more sensitive diagnostic tests for TB will enable the detection of drug resistance among contacts diagnosed with TB [19].

In our study, the NNS over the years was within what has been reported from contact investigation studies conducted in low TB incidence countries [22]. The NNT to identify one TB case in the initial year of our study was comparable to what was reported by Jerene et al. [14]. The observation that in 2010, the year the initiative started, both NNS and NNT were the lowest and the yield of TB cases among the contacts suspected to have TB was the highest may suggest closer adherence to the protocols following the training that kickstarted the initiative.

It can be said that the first component of contact investigation, which consists of contact identification and prioritization, was satisfactorily carried out in the intervention reported on in this paper [3]. It demonstrates the feasibility of successfully listing contacts and screening those suspected to have TB and therefore eligible for testing, as these rates remained high in the intervention years and the year afterwards. As home verification of TB before initiation of treatment is routine practice, as instructed by the NTP, adding contact identification and prioritization was practical and thereby contributed to this achievement.

The same, however, cannot be said for the second component of contact investigation, namely contact clinical evaluation across the period of assessment: there was a noticeable drop of more than 50% in the proportion of presumed positive TB contacts tested/evaluated in the final year of the intervention (2013) and in the following year. The decrease, which could have been due to several reasons, suggests challenges with the steps in the referral system as well as functional laboratory diagnostic services, ranging from limited follow up of presumed TB cases, to ensuring arrival at the microscopy center for testing, to shortages of laboratory diagnostic reagents. This raises concerns of missed opportunities to diagnose TB and the overall effect on the yield of TB cases among the contacts, necessitating further investigation to identify the contributory factors to the decrease.

The End TB Strategy calls for the removal of barriers to seeking care so it is imperative that steps are taken to ensure that presumptive TB cases, who have interacted with the healthcare system, benefit from early and appropriate diagnostic tests as soon as possible to reduce the risk of advanced disease and transmission [17]. Apart from identifying active TB cases, contact investigation can also be a means of preventing disease transmission, as the effective treatment of TB diagnosed among contacts will limit the potential of these persons being a source of infection to others.

With the exception of information on the sex of the TB cases identified among the contacts and the type of TB diagnosed, the database from which the analyses for this study were conducted had no disaggregation by sex for the rest of the measures; consequently there was no information on the sex of the index TB cases nor the contacts listed or evaluated. There was also no data on age for any variable. This limitation precluded the ability to conduct demographic or disaggregated analyses by the type of TB of the index case and especially children, considering that a considerable proportion of childhood TB is diagnosed through household contact investigation [23]. Another limitation of the study is that there was no examination of the possible prevailing factors that accounted for some of the changes in the trends observed in the findings. Despite these limitations, the strength of this paper lies in the documentation of the outcomes of the various components of contact investigation, namely contact identification and prioritization and contact clinical evaluation, over a number of years in a low TB incidence country, where contact investigation has not been routinely practiced.

## Conclusions

This study demonstrates that the contact identification and prioritization components of contact investigation were feasible, but the overall yield of TB cases may have been lower than it could have been if the rate of clinical evaluation of presumptive TB contacts had been sustained over time. Contact investigation as a case finding initiative could be enhanced if actively conducted with the adoption of standardized protocols and sensitive screening tools, training of relevant staff in their use, and removal of barriers to contact screening. Where presumptive TB cases have been identified, it is imperative for the NTP to address possible barriers to accessing recommended sensitive diagnostic tests to enable the maximum yield from contact investigation to be achieved. In the event of scaling up contact investigation, it is also recommended that the NTP widens the scope of the variables in the data collection tools to include minimum key variables such as age (especially children aged under five years), sex, type of TB diagnosed, human immunodeficiency virus (HIV) status of contacts diagnosed with TB and children aged under five years, and persons living with HIV initiated on isoniazid preventive treatment. This will facilitate meaningful analyses of outcomes in specific groups, and subsequent monitoring and tracking of program performance indicators, as per the WHO guidelines.

## Additional file

Additional file 1: Multilingual abstracts in the six official working languages of the United Nations. (PDF 578 kb)

## Abbreviations

AFB: Acid fast bacilli
CIDA: Canadian International Development Agency
DOTS: Directly observed treatment, short-course
DR-TB: Drug-Resistant tuberculosis
EPTB: Extra-pulmonary tuberculosis
HIV: Human immunodeficiency virus
NNS: Number of contacts needed to screen
NNT: Number of contacts needed to test
NTP: National Tuberculosis Control Program
SS + ve: Sputum smear-positive
SS-ve: Sputum smear-negative
TB: Tuberculosis
WHO: World Health Organization.

## References

[1] World Health Organization (WHO). Global tuberculosis report 2016. 2016. http://www.who.int/tb/publications/2016/en/. Accessed 12 Feb 2018.

[2] Morrison J, Pai M, Hopewell PC (2008). Tuberculosis and latent tuberculosis infection in close contacts of people with pulmonary tuberculosis in low-income and middle-income countries: a systematic review and meta-analysis. Lancet Infect Dis.

[3] World Health Organization: Recommendations for investigating contacts of persons with infectious tuberculosis in low and middle-income countries. 2012. http://www.who.int/tb/publications/2012/contact_investigation2012/en/. Accessed 12 Feb 2018. 24404639

[4] Fox GJ, Barry SE, Britton WJ, Marks GB (2013). Contact investigation for tuberculosis: a systematic review and meta-analysis. Eur Respir J.

[5] Ministry of Health (2009). National tuberculosis health sector strategic plan for Ghana 2009–2013.

[6] Bonsu FA, Hanson-Nortey NN, Afutu FK, Kulevome DK, Dzata F, Ahiabu MA (2014). The National Tuberculosis Health Sector Strategic Plan for Ghana 2015–2020.

[7] World Health Organization. WHO-CIDA Initiative: Intensifying TB case detection Update 2011. 2011. http://www.who.int/tb/WHO_CIDA_Initiative_TBUpdate_Ghana.pdf. Accessed 12 Feb 2018.

[8] Ghana Statistical Service (GSS), Ghana Health Service (GHS), and ICF Macro: Ghana Demographic and Health Survey 2008. 2009. http://dhsprogram.com/publications/publication-fr221-dhs-final-reports.cfm. Accessed 12 Feb 2018.

[9] Nair D, Rajshekhar N, Klinton JS, Watson B, Velayutham B, Tripathy JP (2016). Household contact screening and yield of tuberculosis cases —a clinic based study in Chennai, South India. PLoS One.

[10] Volkmann T, Okelloh D, Agaya J, Cain K, Ooko B, Malika T, Burton D (2016). Pilot implementation of a contact tracing intervention for tuberculosis case detection in Kisumu County, Kenya. Public Health Action.

[11] Gashu Z, Jerene D, Ensermu M, Habte D, Melese M, Hiruy N (2016). The yield of community-based “retrospective” tuberculosis contact investigation in a high burden setting in Ethiopia. PLoS One.

[12] Loredo C, Cailleaux-Cezar M, Efron A, de Mello FC, Conde MB (2014). Yield of close contact tracing using two different programmatic approaches from tuberculosis index cases: a retrospective quasi-experimental study. BMC Pulm Med.

[13] Guwatudde D, Nakakeeto M, Jones-Lopez MEC, Maganda A, Chiunda A, Mugerwa RD (2003). Tuberculosis in household contacts of infectious cases in Kampala, Uganda. Am J Epidemiol.

[14] Jerene D, Melese M, Kassie Y, Alem G, Daba SH, Hiruye N (2015). The yield of a tuberculosis household contact investigation in two regions of Ethiopia. Int J Tuberc Lung Dis.

[15] Blok L, Sahu S, Creswell J, Alba S, Stevens R, Bakker MI (2015). Comparative meta-analysis of tuberculosis contact investigation interventions in eleven high burden countries. PLoS One.

[16] Shapiro AE, Variava E, Rakgokong M, Moodley N, Luke B, Saeed Salimi S (2012). Community-based targeted case finding for tuberculosis and HIV in household contacts of patients with tuberculosis in South Africa. Am J Respir Crit Care Med.

[17] World Health Organization: The End TB Strategy: Global strategy and targets for tuberculosis prevention, care and control after 2015. 2014. http://www.who.int/tb/strategy/End_TB_Strategy.pdf?ua=1. Accessed 12 Feb 2018.

[18] Gupta M, Saibannavar AA, Kumar V (2016). Household symptomatic contact screening of newly diagnosed sputum smears positive tuberculosis patients - an effective case detection tool. Lung India.

[19] TB CARE I: International Standards for Tuberculosis Care, 3^rd^ edition 3. 2014. http://www.tbcare1.org/publications/. Accessed 12 Feb 2018.

[20] Singh J, Sankar MM, Kumar S, Gopinath K, Singh N, Mani K (2013). Incidence and prevalence of tuberculosis among household contacts of pulmonary tuberculosis patients in a peri-urban population of South Delhi, India. PLoS One.

[21] World Health Organization: Early detection of tuberculosis: An overview of approaches, guidelines and tools. 2011. http://apps.who.int/iris/handle/10665/70824. Accessed 12 Feb 2018.

[22] Shapiro AE, Chakravorty R, Akande T, Lonnroth K, Golub JE. A systematic review of the number needed to screen to detect a case of active tuberculosis in different risk groups. 2013 http://www.who.int/tb/Review3NNS_case_active_TB_riskgroups.pdf. Accessed 12 Feb 2018.

[23] Ottmani S, Zignol M, Bencheikh N, Laasri L, Blanc L, Mahjour J (2009). TB contact investigations: 12 years of experience in the national TB Programme, Morocco 1993–2004. East Mediterr Health J.

