# Supplementary material for: Yield of tuberculosis among household contacts of tuberculosis patients in Accra, Ghana
Source: Infect Dis Poverty. 2018 Feb 27;7:14. doi: 10.1186/s40249-018-0396-5 (PMC5828410; doi:10.1186/s40249-018-0396-5)
Supplement: Supplementary file 1 — Multilingual abstracts in the six official working languages of the United Nations. (PDF 578 kb) [file 40249_2018_396_MOESM1_ESM.pdf]

Translation of the abstract into the six official working languages of the United Nations

الاختلاط الأسري مع مرضى السل ومردوده على معدل انتشار المرض في آكرا، غانا

سالي آن أوهين، فرانك بونسو، ني نورتي هانسون - نورتي، أديليد ساكي، صامويل دانسو، فيليكس أفوتو، بول كلاتسر، ميريام بكر

الملخص

الخلفية: تدعو استراتيجية القضاء على السل إلى إجراء فحص ممنهج لمجموعات مختارة من المعرضين لخطر الإصابة بدرجة عالية، بما في ذلك المخالطين لمرضى السل لتسهيل الكشف المبكر عن حالات الإصابة بالمرض. لا يخضع المخالطون لمرضى السل للفحص عادةً في الدول ذات معدلات الإصابة المنخفضة بالسل مثل غانا؛ الأمر الذي يترتب عليه ندرة البيانات المتعلقة بحصيلة ما تم اكتشافه من حالات الإصابة بالسل من جراء تلك التدخلات. من ثم، كان الهدف من هذه الدراسة هو توثيق نتائج وجدوى تنفيذ إجراءات فحص المخالطين لمرضى السل وفق ظروف البرنامج في غانا.

المنهج:أُجريت تحليلات بأثر رجعي للبيانات المجردة المستخلصة من البرنامج الوطني لمكافحة السل، بعد التدخل بفحص المخالطين لحالات السل التي تم تشخيصها في 10 مرافق في أكرا من يونيو 2010 حتى ديسمبر 2014. بعد ذلك، تم تقييم النسب المختلفة وحصيلة عدد المخالطين لمرضى السل الذين من المقرر أن يخضعوا للفحص (NNS) وعدد الأشخاص الذين من المقرر أن يخضعوا لاختبار الإصابة بالسل (NNT) بهدف الكشف عن حالات الإصابة بالمرض من بينهم.

النتائج:بوجه عام، تم فحص 8166 حالة (96%) من بين الحالات الواردة في قائمة المخالطين لمرضى السل، وعددهم 8519 حالة من أصل 3627 حالة مدرجة، ووُجِد أن 614 حالة منهم (7.5٪) يشتبه في إصابتها بالسل. ومن بين تلك الحالات، خضعت 438 حالة (71%) للتقييم/ الفحص المجهري لمسحة من البلغم، وتم تشخيص 53 حالة منها. من بين تلك الحالات، كان 56.6٪ من الذكور، و49٪جاءت نتائج فحص السل بمسحة البلغم لديهم موجبة، و38٪ جاءت نتائج فحص السل بمسحة البلغم لديهم سالبة، و7٪ كانوا مصابين بالسل خارج الرئة. وجاء عدد المخالطين لمرضى السل الذين من المقرر أن يخضعوا للفحص (NNS) وعدد الأشخاص الذين من المقرر أن يخضعوا لاختبار الإصابة بالسل (NNT) بهدف الكشف عن حالات الإصابة بجميع أنواع السل 154 و8، على التوالي. كذلك، جاءت نسبة حالات السل بين المخالطين للمدرجين ونسبة المخالطين الذين يخضعون للفحص سنوياً 88 - 96٪ و 83 - 100٪ على التوالي. كما انخفضت نسبة الحالات المشتبه في إصابتها بالسل التي تم اختبارها ونسبة حالات السل التي تم تشخيصها بين المخالطين الذين خضعوا لاختبار الإصابة بالسل إلى 40٪ و 14٪ على التوالي بحلول عام 2014، بعد أن كانت 100٪ و 36٪ على التوالي في عام 2010.

الاستنتاجات:توضح هذه الدراسة أن مقوّمات التعرف على المخالطين لمرضى السل وترتيبهم من حيث الأولوية عند إجراء الفحوص عليهم كانت مجدية. بيد أن الحصيلة الإجمالية لحالات الإصابة بالسل من الممكن أن تكون أقل من ذلك نظراً إلى أن معدل التقييم السريري للمخالطين المشتبه بإصابتهم بالسل ينخفض بمرور الوقت. من ثم، قد يؤدي التصدي للعوائق التي تحول دون الحصول على الاختبارات التشخيصية المناسبة إلى زيادة حصيلة فحوصات المخالطين لمرضى السل في غانا.

Translated from English version into Arabic by Mahmoud Sami, proofread by Heba Kandel, through

TRANSLATORS
WITHOUT BORDERS

加纳阿克拉地区结核病患者家庭接触者的结核病发病率

Sally-Ann Ohene, Frank Bonsu, Nii Nortey Hanson-Nortey, Adelaide Sackey, Samuel Danso, Felix Afutu, Paul Klatser, Mirjam Bakker

摘要

引言：“遏制结核病战略”要求对包括结核病（TB）接触者在内的高危人群进行系统筛查,以便早期发现 TB 病例。在加纳这种 TB 低负担国家很少对接触者进行调查，因此缺乏通过这类干预措施获得 TB 病例检出结果的数据。本研究的目的是记录在加纳国家 TB 项目实施条件下开展接触者调查活动的结果和可行性。

方法：对国家结核病项目的数据进行回顾性分析，随后对阿克拉 10 家医疗机构 2010 年 6 月至 2014 年 12 月间诊断为结核病例的接触者进行调查。对接触者中需要筛查的数量（NNS）和需要检测的数量（NNT），以及相应比例和产出进行评估。

结果：3267 例索引病例共涉及 8519 个接触者，对 8166 人(96%)进行筛查，其中 614 例(7.5%)为疑似结核病例。在这些病例中，438 例(71%)进行了痰涂片镜检，检出 53 例结核病例。其中，56.6%为男性，49%为痰涂片阳性结核病，38%为痰涂片阴性结核病，7%为肺外结核。NNS 和 NNT 中进行 TB 检测的数量分别为 154 和 8。每年有接触者的 TB 比例和筛查接触者的比例分别为 88-96%和 83-100%。2010 年结核病接触者中检出疑似 TB 和诊断为 TB 的比例分别为 100%和 36%，2014 年分别降至 40%和 14%。

结论：本研究表明，接触者调查中的对接触者进行识别和确定优先顺序是可行的，但是随着时间的推移，由于结核病接触者的临床评估率下降，可能检出结核病例数会减少。移除阻碍进行恰当诊断测试的壁垒有助于提高加纳地区 TB 接触者的调查收益。

Translated from English version into Chinese by Translated by Peng Song, edited by Pin Yang

CHINESE CENTER FOR DISEASE CONTROL AND PREVENTION
NATIONAL INSTITUTE OF PARASITIC DISEASES

Taux de tuberculose parmi les contacts familiaux de patients atteints de tuberculose à Accra au Ghana

Sally-Ann Ohene, Frank Bonsu, Nii Nortey Hanson-Nortey, Adelaide Sackey, Samuel Danso, Felix Afutu, Paul Klatser, Mirjam Bakker

Résumé

Rappel des faits: La Stratégie d'éradication de la tuberculose prévoit le dépistage systématique de groupes à haut risque déterminés, y compris les contacts des personnes atteintes de tuberculose pour favoriser la détection précoce des cas de tuberculose. Ce n'est pas pratique courante de faire des recherches sur les contacts dans les pays à faible charge de tuberculose comme le Ghana ; il existe donc peu de données sur le taux de détection de cas de tuberculose à partir de ce type d'interventions. Le but de cette étude était de documenter les résultats et la faisabilité de la réalisation d'activités de recherche sur les contacts dans le cadre d'un programme au Ghana.

Méthodes: On a fait des analyses rétrospectives de données tirées du Programme national sur la tuberculose après une intervention de recherche sur les contacts de cas de tuberculose dans dix établissements à Accra de juin 2010 à décembre 2014. On a évalué diverses proportions et divers taux à partir du nombre nécessaire de contacts à dépister (NND) et du nombre nécessaire à tester (NNT) pour dépister les cas de tuberculose.

Résultats: Dans l'ensemble, sur les 8 519 contacts recensés parmi les 3 267 cas de référence, 8 166 (96 %) ont subi un dépistage et 614 (7,5 %) ont reçu un diagnostic présomptif de tuberculose. Parmi ces contacts, 438 (71 %) ont été soumis à une évaluation ou un examen microscopique de frottis d'expectoration, et 53 cas de tuberculose ont été diagnostiqués. Parmi ces derniers, 56,6 % étaient de sexe masculin, 49 % avaient un frottis d'expectoration positif pour la tuberculose, 38 % avaient un frottis négatif, et 7 % étaient atteints de tuberculose extrapulmonaire. Le NND était de 154 et le NNT de 8 pour dépister un cas de tuberculose, toutes formes confondues. La proportion de cas de tuberculose ayant des contacts recensés était de 88-96 % et la proportion de contacts dépistés annuellement était de 83-100 %. La proportion de cas de tuberculose avec un diagnostic présomptif était de 100 %, et la proportion de cas de tuberculose diagnostiqués parmi les contacts testés était de 36 % en 2010. Ces proportions avaient respectivement chuté à 40 % et à 14 % en 2014.
Conclusions: L'étude montre la faisabilité des aspects d'identification et de priorisation des contacts dans une recherche sur les contacts, mais que le taux d'ensemble des cas de tuberculose était peut-être inférieur en raison de la baisse de fréquence des examens cliniques des contacts avec un diagnostic présomptif de tuberculose au fil du temps. Lever les obstacles à l'accès à des tests diagnostiques pourrait améliorer le taux pour les recherches sur les contacts au Ghana.

Translated from English version into French by Gabrielle Garneau, proofread by Claire V., through

TRANSLATORS
WITHOUT BORDERS

Заболеваемость туберкулезом среди контактных лиц в семейном окружении пациентов с туберкулезом в Аккре, Республика Гана

Sally-Ann Ohene, Frank Bonsu, Nii Nortey Hanson-Nortey, Adelaide Sackey, Samuel Danso, Felix Afutu, Paul Klatser, Mirjam Bakker

Реферат

Основная информация. Для содействия раннему выявлению случаев туберкулеза (ТБ) стратегия «End TB» (Покончить с туберкулезом) призывает к систематическому скринингу отдельных групп высокого риска, включая лиц, имеющих контакт с туберкулезом (ТБ). Обследование контактных лиц не всегда является обычным элементом практики в странах с низким уровнем заболеваемости ТБ, таких как Гана, что приводит к малочисленности данных о частоте выявления случаев ТБ в результате таких интервенций. Целью этого исследования было документальное отражение результатов и возможности внедрения мероприятий по обследованию контактных лиц в Гане согласно программируемым условиям.
Методы. Проводили ретроспективные анализы данных, полученных из Национальной программы по ТБ, с последующей интервенцией в виде обследования контактных лиц для случаев ТБ, диагностированных в 10 учреждениях в Аккре с июня 2010 г. по декабрь 2014 г. Проводили оценку различных соотношений и показателей заболеваемости, используя

количество контактных лиц, нуждающихся в скрининге (КНС), и количество лиц, нуждающихся в тестировании (КНТ).
Результаты. Всего из 8 519 людей, указанных в списке как контактные лица с 3 267 источниками заболевания, скрининг провели у 8 166 (96%) людей, из них у 614 (7,5 %) установлен предварительный диагноз ТБ. Микроскопию/изучение мазка мокроты провели у 438 (71%) из 614 людей, и ТБ диагностирован у 53. Среди этой группы (53 человека) 56,6 % были мужчинами, у 49% был положительный результат мазка мокроты на ТБ, у 38% был отрицательный результат мазка мокроты на ТБ, и у 7% был обнаружен внелегочный ТБ. Показатели КНС и КНТ для выявления ТБ в любой форме составляли 154 и 8 соответственно. Соотношение случаев ТБ с указанными контактными лицами и соотношение проводимого скрининга контактных лиц ежегодно составляло 88-96% и 83-100% соответственно. Соотношение людей с предварительным диагнозом ТБ, прошедших тестирование, и соотношение случаев ТБ, диагностированных у контактных лиц, в 2010 г. составляло 100% и 36% соответственно, а к 2014 г. показатели снизились до 40% и 14%.
Выводы. Исследование показывает возможность как выявления контактных лиц, так и приоритизации компонентов их обследования, однако общий показатель заболеваемости ТБ возможно был меньше, принимая во внимание снижение частоты клинического обследования контактных лиц с предварительным диагнозом ТБ на протяжении указанного отрезка времени. Показано, что при нацеленных на преодоление барьеров усилиях для оценки соответствующих диагностических тестов можно добиться увеличения показателей выявления ТБ у контактных лиц в Гане.

Translated from English version into Russian by Ann Nosova, proofread by tatiana_com, through

TRANSLATORS
WITHOUT BORDERS

Incidencia de la tuberculosis entre contactos domésticos de pacientes con tuberculosis en Accra, Ghana

Sally-Ann Ohene, Frank Bonsu, Nii Nortey Hanson-Nortey, Adelaide Sackey, Samuel Danso, Felix Afutu, Paul Klatser, Mirjam Bakker

Resumen
Introducción: La Estrategia Fin a la Tuberculosis pide la detección sistemática de determinados grupos de alto riesgo como los casos de contactos de tuberculosis (TB) para facilitar la detección temprana de TB. La investigación de contacto a menudo no se practica de forma rutinaria en países con baja carga de TB como Ghana, esto lleva a una escasez de datos sobre la incidencia de la detección de casos de TB en tales intervenciones. El objetivo de este estudio era documentar los resultados y la viabilidad de implementar actividades de investigación de contacto bajo condiciones programáticas en Ghana.

Métodos: Se realizaron análisis retrospectivos de los datos resumidos del Programa Nacional de TB, seguidos de intervenciones de investigación de contacto para casos de TB diagnosticados en 10 instalaciones en Accra de junio de 2010 a diciembre de 2014. Fueron evaluadas varias medidas e incidencias del número de contactos necesarios a detectar (NND) y del número de contactos necesarios a examinar (NNE) para descubrir casos de TB.

Resultados: En total, de los 8519 contactos listados con 3267 casos índice, 8166 (el 96%) fueron detectados y 614 (el 7,5%) fueron identificados con presunta TB. De estos contactos, a 438 (el 71%) se les practicó un frotis de esputo/investigación bacterioscópica y se diagnosticaron 53 casos de TB. De estos casos, el 56,6% eran hombres; el 49% tuvo resultados positivos de TB con el frotis de esputo, el 38% tuvo resultados negativos de TB en el análisis de esputo y al 7% se le diagnosticó TB extrapulmonar.

Los NND y NNE que detectaron casos de TB de todo tipo fueron 154 y 8, respectivamente. La proporción de casos de TB con contactos listados y la proporción de contactos detectados anualmente fueron del 88 al 96% y del 83 al 100%, respectivamente. La proporción de casos examinados de presunta TB y la proporción de casos diagnosticados de TB entre los contactos examinados que en el 2010 fueron del 100% y 36%, respectivamente, cayeron al 40% y al 14%, respectivamente en el 2014.

Conclusiones: El estudio demuestra que la identificación y priorización del contacto como componentes de la investigación del contacto eran viables, pero la prevalencia de los casos de TB pueden haber bajado debido a la disminución de la tasa de la evaluación clínica de presunta TB de contactos con el tiempo. Hacer frente a los obstáculos para acceder a pruebas de diagnóstico apropiadas puede mejorar el rendimiento de la investigación de contacto en Ghana.

Translated from English version into Spanish by SaraiDLP, proofread by sylfigu, through

TRANSLATORS
WITHOUT BORDERS
